# Progress on the prevention of poultry Salmonella with natural medicines

**DOI:** 10.1016/j.psj.2024.104603

**Published:** 2024-11-27

**Authors:** Yi Zhang, Jianglan Liu, Yinan Pan, Kai Shi, Ping Mai, Xiaokai Li, Shasha Shen

**Affiliations:** Institute of Laboratory Animal Sciences, Panzhihua University, Panzhihua 617000, China

**Keywords:** Poultry Salmonella, Natural medicines, Antibiotics

## Abstract

*Salmonella* infection is an acute and systemic disease of poultry, primarily affecting young birds. The mortality rate of chicken within one week of age can reach up to 40 %. Surviving individuals may become carriers of the bacteria, leading to latent infections that can result in bacterial residues in meat and egg products, posing serious threats to human food safety and health. Antibiotic therapy is one of the most conventional treatments for *Salmonella* infections in birds. However, the current abuse of antibiotics has accelerated the mutation of pathogenic bacteria to generate antibiotic-resistant strains. Thus, the effectiveness of treatment with antibiotics alone is gradually diminishing. To address this threat, researchers have explored the use of natural products to enhance the immune system of poultry for preventing *Salmonella* infections. This study aims to provide a comprehensive review, systematically summarizing recent research progress of the application of natural medicines on poultry *Salmonella* infection.

## Highlights

• Globally, the number of people infected with *Salmonella* through food can reach 115 million per year. Immunocompromised populations such as children (especially those under 5 years of age), the elderly, and pregnant women are particularly susceptible.
• Antibiotic treatment, although effective in controlling *Salmonella* in poultry, carries a very high risk of environmental pollution.
• In this article, we reviewed the advanced strategies of natural medicines against *Salmonella* infection.
• We comprehensively illustrated the research progress and antibacterial mechanisms of natural medicines such as probiotics, prebiotics, postbiotics, phytobiotics, essential oils, water-soluble plant extracts, and traditional Chinese medicine compounds.
• Incorporated with cutting-edge drug delivery methods such as in ovo injection and nanobiomaterials, we proposed the development directions of potential natural anti-*Salmonella* drugs for poultry.

## Introduction

**Fig. 1:**
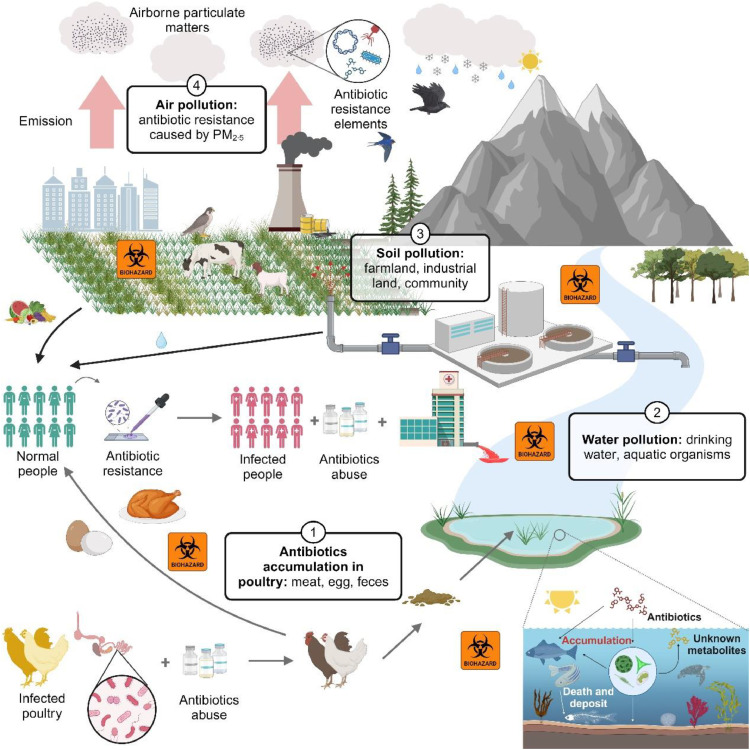
Environmental pollution caused by abuse of avian antibiotics. Past clinical guidelines for the use of large quantities of antibiotics as additives to poultry feed and water for the control of Salmonella in poultry have resulted in the deposition of antibiotics into poultry meat, eggs, and feces. Antibiotics in feces are further deposited into soil and rivers, leading to river pollution and enrichment in crops and aquatic organisms. Consumption of food or drinking water containing excessive antibiotic residues induces antibiotic resistance in the body. Infected individuals with antibiotic resistance may require further high-dose antibiotic treatments in hospitals to control acute symptoms, increasing the burden on their bodies. The resistance elements can be emitted into the environment through medical waste, domestic and industrial waste, and even airborne particulates. This environmental pollution further triggers a vicious cycle and leads to poultry antibiotic resistance. Adapted with permission from BioRender. Kelai, W. (2024).

*Salmonellosis* is a common bacterial disease that affects the intestinal tract in humans, and is one of the four key global causes of diarrheal diseases as identified by the World Health Organization (Havelaar et al., 2015). Poultry meat and eggs are important carriers of *Salmonella* (Havelaar et al., 2015). To counter the threat posed by *Salmonella* in poultry production, antibiotic therapy is frequently recommended for clinical control (Suresh et al., 2018). Antibiotics start working fast and significantly shorten the duration of illness (Suresh et al., 2018). However, uncontrolled antibiotic usage not only diminishes their effectiveness but also results in the presence of residual antibiotics in poultry. Research has identified that domestic egg products can contain residues of over 20 types of antibiotics, including tetracycline and chloramphenicol, which pose serious health risks to humans (Ben et al., 2022; Lima et al., 2023; Ma et al., 2022; Mund et al., 2017; Wang et al., 2022; Yang et al., 2020). Excessive antibiotics in the poultry gut are excreted through feces, contaminating the surrounding environment, including soil, water, and even the air (Muhammad et al., 2020). Long-term consumption of contaminated food and water destroys the balance of intestinal flora, stimulates the bacteria to evolve new antibiotic-resistant strains, and reduces the effectiveness of antibiotic treatments (Fig. 1). When the body is invaded by high-risk pathogens, such as those causing pneumonia, tuberculosis, sepsis, and gonorrhea, treatment becomes difficult or even impossible (Huemer et al., 2020). According to statistics from the U.S. Centers for Disease Control and Prevention (CDC), approximately 200,000 people in the United States die annually from antibiotic-resistant infections (Kadri, 2020). To circumvent this vicious cycle and eliminate antibiotic residues in poultry products, in recent years, many scholars have proposed the use of natural drugs to replace antibiotics in the treatment of poultry diseases. Numerous pharmacological analyses and clinical practices have proved that probiotic and plant-derived drugs have great potential in the control of poultry *Salmonella* (Chen et al., 2022; Khan and Chousalkar, 2020; Reda et al., 2021; Wang et al., 2021; Wu et al., 2020). This article seeks to review and consolidate the research findings in this domain, explore the trajectory toward achieving sterile and green poultry food production, and foster the healthy and sustainable development of the poultry breeding industry.

## Mechanism of Salmonella pathogenesis

### Poultry Salmonella strains

*Salmonella* is a Gram-negative, facultatively anaerobic bacterium belonging to the family Enterobacteriaceae. It primarily includes two species: *Salmonella Enterica* and *Salmonella Bongori*. So far, more than 2500 serotypes belonging to the genus *Salmonella* have been described worldwide, many of which are capable of causing disease in humans and animals (Berhanu and Fulasa, 2020). Despite their similarities in genomic sequences, these *Salmonella* serotypes exhibit significant differences in their distribution across various host types and host organs (Berhanu and Fulasa, 2020). The *Salmonella Enterica* serotypes specific to poultry include host-specific pathogens such as *Salmonella Gallinarum* and *Salmonella Pullorum*, which respectively cause typhoid and pullorum disease in chickens (Berhanu and Fulasa, 2020; Saif, 2009; Zhou et al., 2022). A meta-epidemiological analysis of 201 global poultry qualitative studies (covering over 900 million samples) conducted between 1945 and 2021 showed a prevalence rate (the percentage of positive samples in the total sample) of 8.54 % (95 % CI: 8.43–8.65) for *Salmonella* in chickens, with a V-shaped growth trend over time (Zhou et al., 2022). *Salmonella Pullorum* infections are particularly prevalent in Asia, especially in eastern China. Epidemiological surveys indicate that the incidence of pullorum disease in China can reach 43.9 % (Lv et al., 2022; Talukder et al., 2023; Wang et al., 2020). The carriage rate of subclinical *Salmonella Pullorum* in healthy chicken flocks can be as high as 46.5 % (Lv et al., 2022; Talukder et al., 2023; Wang et al., 2020). Additionally, *Salmonella Pullorum* can co-infect chicken flocks with other *Salmonella* strains, such as *Salmonella Gallinarum* and *Salmonella Typhimurium*, thereby broadening the host range and increasing the risk of foodborne typhoid fever and diarrhea in humans (Lv et al., 2022; Talukder et al., 2023; Wang et al., 2020).

### Transmission of poultry Salmonella

The transmission routes of poultry *Salmonella* can be divided into two main types: 1) horizontal transmission and 2) vertical transmission. Horizontal transmission is the primary route of *Salmonella* spread in poultry (Berhanu and Fulasa, 2020). Weak or newborn chicks can be infected with *Salmonella* by contact with diseased feces, contaminated feed, water, and bedding (Berhanu and Fulasa, 2020; Saif, 2009; Tariq et al., 2022). Infected chickens become intestinal carriers, and over a certain period, pathogens are excreted from their bodies through feces, thereby facilitating horizontal transmission, which can ultimately lead to large-scale outbreaks of typhoid or pullorum disease (Berhanu and Fulasa, 2020; Saif, 2009; Tariq et al., 2022). Therefore, contaminated feces are a great threat to uninfected chickens. The probability of disease outbreaks of poultry *Salmonella* infections tends to be higher in farms with poor environmental hygiene and highly intensive rearing. Besides the digestive tract, the horizontal transmission may also occur through skin wounds, blood, mating, contact with reproductive secretions, or the use of contaminated transportation equipment (Berhanu and Fulasa, 2020; Saif, 2009; Tariq et al., 2022).

In contrast, the vertical transmission mode of *Salmonella* in poultry has always been a subject of debate. *Salmonella* can infect the reproductive organs and its strains can be detected in the eggs of infected birds. However, newborn chicks can survive normally and even exhibit resistance to *Salmonella* (Berhanu and Fulasa, 2020). Despite not affecting survival, these offspring chicks became long-term carriers of *Salmonella*. Maternally derived *Salmonella* strains are still reported to be detectable in the feces of 14-week-old vertical progeny flocks (Berhanu and Fulasa, 2020). This innate resistance is not present in horizontally infected flocks. Epidemiological studies show that both *Salmonella Gallinarum* and *Salmonella Pullorum* have high morbidity and mortality rates. Mortality within two weeks in chicks horizontally infected with *Salmonella Pullorum* can even reach 100 %, and mortality in chicks infected with *Salmonella Gallinarum* can reach 50 % (Berhanu and Fulasa, 2020; Lv et al., 2022; Talukder et al., 2023; Wang et al., 2020; Zhou et al., 2022). In contrast, the survival rate of offspring flocks with vertical transmission can exceed 90 % (Berhanu and Fulasa, 2020; Lv et al., 2022; Talukder et al., 2023; Wang et al., 2020; Zhou et al., 2022). The presence of maternal antibodies and immune factors may restrict the virulence of vertically transmitted *Salmonella*, causing it to transform into an "attenuated" strain. This weakened variant may produce a biological effect akin to vaccination, helping vertically transmitted offspring to form their own *Salmonella* immune barrier during growth.

## Clinically used broad-spectrum antibiotics and their limitations

Antibiotics commonly used in the treatment of poultry *Salmonella* infections include enrofloxacin, ciprofloxacin, azithromycin, ceftriaxone, chloramphenicol, neomycin, polymyxin, nitrofurazolidone, amoxicillin, tetracycline, etc. (Farhat et al., 2023; Ruvalcaba-Gómez et al., 2022; Shaji et al., 2023). Given the rapid action of antibiotics, which can effectively reduce the mortality rate of poultry infected with *Salmonella* during the acute phase, they are still widely used as the primary clinical treatment for *Salmonella* infections in poultry (Farhat et al., 2023; Ruvalcaba-Gómez et al., 2022; Shaji et al., 2023). In some countries and regions, antibiotics such as penicillin, tetracycline, and chloramphenicol are also added to poultry feed to prevent *Salmonella* contamination, thereby improving feed conversion rates, enhancing poultry growth performance, and inhibiting pathogens within the gut (Farhat et al., 2023; Ruvalcaba-Gómez et al., 2022; Shaji et al., 2023).

Antibiotic prevention and control strategies have good clinical practice. However, its potential problems and hazards still cannot be ignored. The main limitations include the following: 1) Antibiotic resistance. Abuse of antibiotics will accelerate the mutation and evolution of *Salmonella*, forming new drug-resistant flora and exacerbating the difficulty of *Salmonella* treatment and prevention (Fig. 1) (Huemer et al., 2020; Lima et al., 2023; Muhammad et al., 2020; Mund et al., 2017; Yang et al., 2020; Zhou et al., 2023); 2) Antibiotic pollution. Antibiotics and their metabolites will remain in poultry feces and then excreted, contaminating soil and water, affecting the growth of plants and livestock and poultry, and ultimately enriched in the human body, causing abnormal reactions (Fig. 1) (Huemer et al., 2020; Lima et al., 2023; Muhammad et al., 2020; Mund et al., 2017; Yang et al., 2020; Zhou et al., 2023); 3) potential drug safety issues. The abuse of antibiotics has associated drug safety concerns. It may disrupt the balance of gut microbiota and inhibit immune cell function, leading to inflammation, allergies, and even irreversible hepatic and renal injury (Clifford et al., 2022; Huemer et al., 2020; Lima et al., 2023; Muhammad et al., 2020; Mund et al., 2017; Park et al., 2021).

## Advantages of natural medicines

**Fig. 2:**
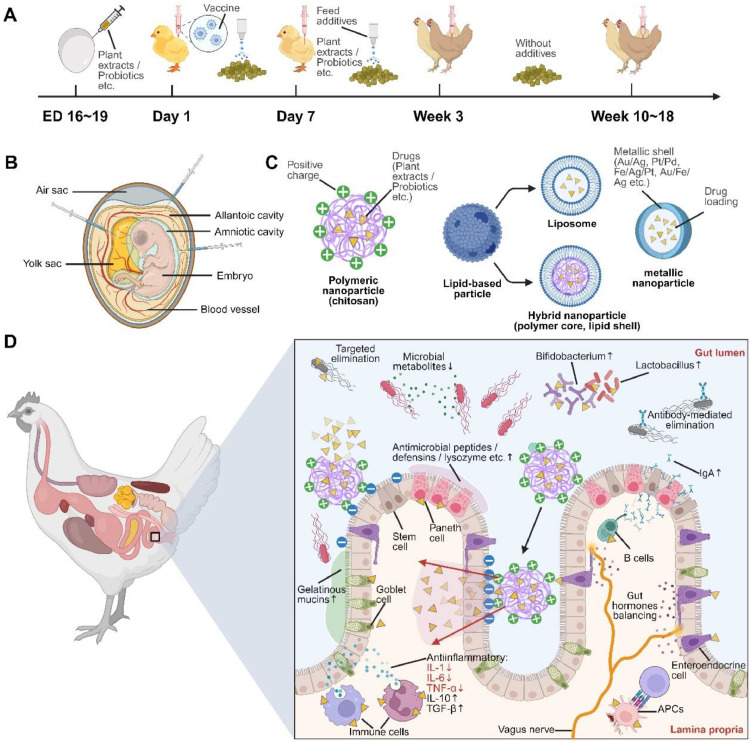
Clinical application and antimicrobial mechanism of anti-Salmonella avian natural drugs. A) The process of combined application of Salmonella avian vaccine and natural drugs; B) The basic method of intravitreal injection of Salmonella avian vaccine or natural drugs; C) Types and structure of natural drug nano-delivery systems; and D) Mechanisms of nanoparticles encapsulated with natural drugs to fight against Salmonella avian infections. The active ingredients in natural drugs can alter the permeability of Salmonella avian biofilm and interfere with the bacterial community sensing mechanism, thus directly killing or inhibiting the proliferation of Salmonella avian. The chitosan nanoparticles used in the encapsulated natural drug carry a positive charge on the surface, which can be in close contact with the negatively charged gastrointestinal mucosal layer, ensuring that the drug is effectively delivered to the intestinal epithelium and penetrates into the intestinal lamina propria. The natural drug stimulates the immune cells in the lamina propria to release anti-inflammatory factors, relieves Salmonella-induced intestinal inflammation, improves the efficiency of antigen presentation, and directs the body's cellular and humoral-mediated immune response to remove bacteria from intestinal tissues and lumen. Natural medicines can also activate intestinal mucosal Pan cells, cup cells, and secretory cells, promote the secretion of antibacterial components such as inhibitory peptides, defensins, lysozyme, and colloidal mucin, balance the synthesis of intestinal hormones, and regulate the intestinal absorption of nutrients. ED, time of incubation (day); APCs, antigen-presenting cells. Adapted with permission from BioRender. Kelai, W. (2024).

Natural medicines offer several benefits, including low toxicity, high safety, efficient metabolism, and no environmental pollution, effectively compensating for the shortcomings of antibiotics (Table 1 and Fig. 2). Unlike antibiotics, the active ingredients in natural medicines, such as unsaturated fatty acids, proteins, polysaccharides, and alkaloids, can be utilized or metabolized by host animals and gut microbiota. This not only significantly alleviates the metabolic burden on the liver and kidneys but also prevents environmental pollution caused by residual drug components. During the clearance of *Salmonella*, natural medicines enhance the richness of gut beneficial microbes. Their relatively mild bactericidal effect is conducive to the recovery of infected animals after the acute phase. Excessive antibiotics will inhibit the phagocytic activity of macrophages and responses mediated by other immune cells (Rowe et al., 2020; Saavedra et al., 2024). These adverse reactions are rare to be observed in the application of natural medicines. Compared with antibiotics, the antimicrobial mechanisms of natural medicines are more diverse (Table 1 and Fig. 2). The active ingredients in natural drugs can alter the permeability of *Salmonella* biofilm and interfere with the bacterial community sensing mechanism to directly kill or inhibit the proliferation of *Salmonella* (Abd El-Hack et al., 2022; Angane et al., 2024; El-Saadony et al., 2022; Ruvalcaba-Gómez et al., 2022; Shaji et al., 2023). They can also activate intestinal mucosal Pan cells, cup cells, and secretory cells, promote the secretion of antibacterial components such as inhibitory peptides, defensins, lysozyme, and colloidal mucin, balance the synthesis of intestinal hormones, and regulate the intestinal absorption of nutrients (Bialkowski et al., 2023; Huang et al., 2024; Spisni et al., 2020). In addition, natural medicines stimulate the immune cells in the lamina propria to release anti-inflammatory factors, relieve *Salmonella*-induced intestinal inflammation, improve the efficiency of antigen presentation, and direct the cellular and humoral-mediated immune response to remove bacteria from intestinal tissues and lumen (El-Sayed et al., 2024; Liu et al., 2023, 2024). Considering these advantages, increased applications of natural medicines against *Salmonella* infection in poultry have been reported (Alves et al., 2024; Chen et al., 2022; Groves et al., 2021; Hu et al., 2023; Kačániová et al., 2024; Shen et al., 2024). There are two main categories of popularized natural medicines: 1) probiotics and prebiotics; 2) plant extracts, such as phytobiotics and essential oils. Their details are introduced in the following sections.

**Table 1:** Clinical application of natural medicines against poultry *Salmonella*.

**Natural medicine**	**Types**	**Target species**	**Dosing time**	**Delivery methods**	**Duration**	**Challenge time**	**Results**	**References**
*Bacillus subtilis* DSM 32324, *Bacillus subtilis* DSM 32325, and Bacillus *amyloliquefaciens*	Probiotics	Isa-Brown layer hens	Week 14	Oral feeding / feed additives	4 weeks	Week 18	Probiotic supplementation decreased *Salmonella* counts, and increased the levels of butyrate and propionate in feces.	(Khan and Chousalkar, 2020)
*Lactobacillus salivarius* Erya	Probiotics	Arbor Acres broilers	Day 1	Oral feeding / feed additives	2 weeks	Week 2	*Lactobacillus salivarius* could degrade AFB1, and enhance *Salmonella Pullorum* infection resistance in broilers challenged with AFB1.	(Chen et al., 2022)
Trehalose dihydrate	Prebiotics	Arbor Acres broilers	Day 1	Oral feeding / water	5 weeks	Week 4	Trehalose supplementation alleviated the adverse effects on gastrointestinal system from *Salmonella Typhimurium* challenge, increased the abundance of *lactobacilli*, and suppressed the growth and inflammation caused by *Salmonella Typhimurium*.	(Wu et al., 2020)
Beta levaFructan, *Lactobacillus acidophilus, Lactobacillus plantarum, Pediococcus pentosaceus, Saccharomyces cerevisiae, Bacillus subtilis, Bacillus licheniformis*	Probiotics and prebiotics	Cobb broilers	Day 1	Oral feeding / water	2 weeks	Week 2	Probiotic and prebiotics could diminish the negative effect of live vaccine on growth performance, and decrease the fecal isolation of *Salmonella Enteritidis*.	(El-Shall et al., 2019)
*Enterococcus faecium, Pediococcus acidilactici, Bifidobacterium animalis, Lactobacilus reuteri*, fructooligosaccharides, vaccine	Synbiotics and polysaccharides	Hyline brown egg layers	Day 1	Oral feeding / water and feed additives	16 weeks	Week 17	The synbiotics augmented the vaccine's protective capacity to limit the re-excretion of *Salmonella Typhimurium* at sexual maturity and decrease the susceptibility to subsequent challenge.	(Groves et al., 2021)
*Lactobacillus rhamnosus* HN001, *Pediococcus acidilactici* MA18/5M, Agave tequilana fructans	Synbiotics	COBB Avian48 broilers	Day 1	Oral feeding / water	38 days	Day 1	Synbiotics supplementation influenced morphological modifications in the duodenal mucosa, inhibited the growth of *Salmonella Typhimurium* and *Clostridium perfringens*, and decreased the intensity and frequency of histopathological injuries.	(Villagrán-de la Mora et al., 2019)
Maltodextrin, Potato extract dextrin, Iodinated casein	Prebiotics	Commercial broilers’ hatching eggs	ED 18.5-19	In-ovo injection / yolk sac	24 h	Day 1	The iodinated casein in combination with dextrin improved the hatchability and early growth. But no differences in *Salmonella Enteritidis* colonization after challenging.	(Abousaad et al., 2017)
*Enterococcus faecium* and *Bacillus subtilis*	Probiotics	Ross 308 broiler’ hatching eggs	ED 17.5	In-ovo injection / amniotic fluid	24 h	Day 4	In-ovo inoculating probiotics significantly reduced the number of *Salmonella Enteritidis* positive chicks.	(De Oliveira et al., 2014)
Commercial phytobiotics Intebio consists of a carrier (wheat bran, GOST 7169-66) enriched with a mixture of essential oils (derived from garlic, lemon, thyme and eucalyptus)	Phytobiotics and essential oils	Ross 308 broilers	Day 1	Oral feeding / feed additives	18 days	Day 19	Intebio upregulated the gene expression of AvBD10, IL6, IL8L2, CASP6, and IRF7 at day 1 post infection, and lowered them at day 23. Intebio promoted response to infection and induced an earlier suppression of the inflammatory reaction in the early infection stage.	(Laptev et al., 2019)
Commercial phytobiotics of 7 plant extracts (oregano, eucalyptus, thyme, garlic, lemon, rosemary, and sweet orange)	Phytobiotics and essential oils	Ross 308 broilers	Day 1	Oral feeding / feed additives	5 weeks	Day 15	Dietary supplementation with phytobiotics could effectively compare with the antibiotic avilamycin in the maintenance of growth performance and improvement in meat characteristics of broilers challenged with *Salmonella Typhimurium*.	(Aljumaah et al., 2020)
Commercial blend product of coated essential oils and organic acids (thymol, carvacrol, cinnamaldehyde, caprylic acid, benzoic acid, butyric acid)	Phytobiotics and essential oils	Arbor Acres broilers	Day 1	Oral feeding / feed additives	6 weeks	Day 13	Dietary supplementation with essential oils significantly reversed the negative effects caused by *Salmonella Enteritidis* infection, increased the gene expression of CLDN-1, OCLN, ZO-1, MUC-2, FABP-2, NF-κB, MyD88, IL-6, but decreased the expression of TLR-4.	(Hu et al., 2023)
Alfalfa polysaccharides	Polysaccharides	Arbor Acres broilers	Day 1	Oral feeding / feed additives	6 weeks	Day 1	Supplementation with Alfalfa polysaccharide enhanced the richness of gut beneficial microbes, while decreased the abundance of facultative anaerobic bacteria.	(Li et al., 2021b)
Glycyrrhiza polysaccharides	Polysaccharides	Japanese quails	Week 1	Oral feeding / feed additives	5 weeks	Maternal transmission	Glycyrrhiza polysaccharides supplementation resulted in marked reductions in the number of total bacteria, coliforms, *Escherichia coli*, and *Salmonella*, compared to those in the control.	(Reda et al., 2021)
*Curcuma longa, Houttuynia cordata, Prunus mume, Rubus coreanus*	Herb mixture	Ross 308 broilers	Day 20	Oral feeding / feed additives	2 weeks	Day 35	Herb mixture potentially stimulated the nonspecific immune responses in broilers and increased the survival ability against *Salmonella Gallinarum*.	(Jung et al., 2010)
*Astragalus, P. notoginseng*, licorice, chickpeas, black bean powder and glucose	Herb mixture	Nongda No. 3 Dwarf chicks	Day 1	Oral feeding / water and feed additives	7 days	Day 1	Herb mixture significantly improved the growth performance of the thymus and bursa, regulated intestinal flora, enhanced immune ability, and prevented death in *Salmonella pullorum* infected chicks.	(Wang et al., 2021)

Duration: duration of natural medicine administration; challenging time: time of *Salmonella* challenging; ED: time of incubation (day).

## Probiotics and prebiotic group medicines

### Probiotics

Probiotics and prebiotics play an important role in promoting intestinal health and supporting the balance of beneficial intestinal flora in poultry (Table 1 and Fig. 2). Probiotics provide beneficial effects to the host through the competitive exclusion of harmful bacteria, modulating the immune system, and improving digestive and absorption functions in the gut, thus promoting the growth performance of chicken (Ruvalcaba-Gómez et al., 2022; Shaji et al., 2023). The microorganisms used as probiotics in poultry supplementation include spore-forming *Bacillus subtilis*, yeast species from the *Saccharomyces* genus, as well as species from the *Enterococcus, Streptococcus, Lactobacillus*, and *Bifidobacterium* genera. The addition of probiotic supplements to the diet of *Salmonella*-infected laying hens increases the accumulation of short-chain fatty acids (SCFAs) (acetic, butyric, and propionic acids) in the gut and blood, which helps to restore the capacity of the post-infectious gut microflora (Ruvalcaba-Gómez et al., 2022; Shaji et al., 2023). Probiotics also produce antimicrobial compounds, such as hydrogen peroxide, lactic acid, bacteriocins, and SCFAs, which inhibit the proliferation or colonization of *Salmonella* in target organs (e.g., cecum) (Ruvalcaba-Gómez et al., 2022; Shaji et al., 2023). Furthermore, probiotics can enhance the efficacy of *Salmonella* vaccines used in poultry, strengthening the preventive effect of the body's immune barriers.

### Prebiotics

Prebiotics are a class of selective compounds that cannot be absorbed or metabolized by the body but can be utilized by gut microbiota, particularly beneficial bacteria (Table 1 and Fig. 2). They play an important role in promoting gut health and supporting the balance of beneficial gut flora in poultry. Prebiotics commonly used for the prevention and control of *Salmonella* in poultry are primarily derived from indigestible oligosaccharides and polysaccharides of yeast cell walls, such as mannan-oligosaccharides, fructo-oligosaccharides, inulin, and xylo-oligosaccharides (Ruvalcaba-Gómez et al., 2022; Shaji et al., 2023). These prebiotics have been shown to inhibit the survival and colonization of pathogens like *Salmonella* in poultry by producing SCFAs, such as butyric acid and acetic acid, in the cecum, which lowers the intestinal pH, while also promoting the proliferation of beneficial gut bacteria (Ruvalcaba-Gómez et al., 2022; Shaji et al., 2023). Studies have reported that adding polysaccharides derived from yeast cell walls to the diet of laying hens reduced *Salmonella* pathogen counts in feces and intestines, increased the production of the anti-inflammatory cytokine IL-10, and enhanced the proliferation of beneficial bacteria like *Lactobacillus* in the cecal tonsils and their fermentation by-products (Ruvalcaba-Gómez et al., 2022; Shaji et al., 2023). Further research found that yeast cell prebiotics effectively increase the percentage of regulatory T cells, thereby regulating the expression of the anti-inflammatory IL-10 gene in the cecal tonsils, reducing the expression of the pro-inflammatory IL-1 gene, and improving the growth performance of broilers (Ruvalcaba-Gómez et al., 2022; Shaji et al., 2023). Similar studies have shown that supplementation of broiler feed with 5 % alginate or 1 % fructooligosaccharides effectively improved feed utilization, promoted the growth of lactobacilli in the duodenum and jejunum, up-regulated the expression of the TLR4 receptor, increased the IgA antibody titer in ileal cells after *Salmonella Gallinarum* infection, inhibited the reproduction rate of pathogenic bacteria, decreased the load on the cecum, and reduced inflammatory responses in the intestinal tract (Ruvalcaba-Gómez et al., 2022; Shaji et al., 2023).

### Synbiotics

Synbiotics refer to combined formulations of probiotics and prebiotics. However, synbiotics are not a random combination of probiotics and prebiotics, but rather a combination of certain probiotics that can colonize the intestines and prebiotics that can be specifically utilized by these probiotics (Table 1 and Fig. 2). Compared with the use of prebiotics or probiotics alone, this combination is more conducive to the proliferation and fermentation of probiotics in the intestinal tract. They produce SCFAs and amplify the effects on the improvement of the gut micro-ecological environment, active the host's immune functions, and inhibit the translocation of pathogenic bacteria (Ruvalcaba-Gómez et al., 2022; Shaji et al., 2023). Studies have shown that the supplementation of synbiotics can improve the production performance of breeding poultry. Individuals fed synbiotics exhibited less heat stress compared to chickens fed regular diets (Ruvalcaba-Gómez et al., 2022; Shaji et al., 2023). Additionally, synbiotics effectively control the impact of *Salmonella* on laying performance. Dietary supplementation with synbiotics improves body weight gain in laying hens, reduces *Salmonella* intestinal colonization, regulates the function of lymphoid organs (suprascapular glands, spleen), increases the size of bursal follicles and stimulates the secretion of immunoglobulins, which improves the immune competence against *Salmonella* infection in laying hens, and prevents the drop in egg production caused by *Salmonella* infection (Ruvalcaba-Gómez et al., 2022; Shaji et al., 2023).

### Postbiotics

Postbiotics, also known as “biogenics”, are soluble factors secreted by the metabolic activity of living bacteria (metabolites) or released by bacterial death and lysis, which are capable of exerting beneficial effects on the host (Table 1 and Fig. 2). Clinically used postbiotics include SCFAs, enzymes, peptides, phosphoglycans, peptidoglycan derivatives, endogenous and exogenous polysaccharides, bacterial exomembrane proteins, vitamins, bile acids, acetylated phospholipids, and long-chain fatty acids (Ruvalcaba-Gómez et al., 2022; Shaji et al., 2023). Although postbiotics themselves are not live bacteria, their mode of action on host metabolism through soluble factors is similar to that of probiotics. The benefits of these postbiotics to the host are diverse, including immunomodulatory effects, lowering intestinal pH, suppressing intestinal pathogens (pathogen antagonism), boosting antioxidant activity, promoting gut health, safeguarding barrier function, and enhancing performance (Ruvalcaba-Gómez et al., 2022; Shaji et al., 2023). Experimental evidence confirms that oral administration of postbiotics significantly reduced *Salmonella*-related infections in poultry. Oral administration of the postbiotic Albusin B increased the expression of PPET1 (endothelin 1 precursor protein) in the jejunum of broiler chicks, enhanced glucose and protein absorption in the intestine, and inhibited *Salmonella* adhesion and colonization using its lectin structural domain (Ruvalcaba-Gómez et al., 2022; Shaji et al., 2023). Similarly, dietary supplementation with postbiotic RG14 (0.15 % and 0.45 %) and the prebiotic inulin (1 %) improved body weight in poultry, increased the proportion of *Bifidobacterium* in the cecum, and reduced the expression of interleukin LITAF (lipopolysaccharide-induced tumor necrosis factor alpha) and interferon IFN genes (Ruvalcaba-Gómez et al., 2022; Shaji et al., 2023). In addition, as the most commonly used postbiotic―lactobacillus bacteriocins, rich in arginine, lysine, and histidine, have cationic properties in a neutral pH environment, which endows them with the ability to bind pathogens and disrupt their cellular integrity, which effectively reduces the signs of *Salmonella* infections and significantly reduces morbidity and mortality (Ruvalcaba-Gómez et al., 2022; Shaji et al., 2023).

## Plant extract-based medicines

### Phytobiotics

Phytobiotics are bioactive compounds extracted from plants that have broad-spectrum antibacterial effects and are considered potential alternatives to traditional antibiotics for feed additives or clinical disease control. Saponins, flavonoids, terpenes, and alkaloids extracted from plants, all of which possess antibacterial properties, are categorized as plant bio-antibiotics (Angane et al., 2024; Ruvalcaba-Gómez et al., 2022; Sakarikou et al., 2020; Shaji et al., 2023). These compounds possess long-lasting antioxidant, antiviral, antimicrobial, anticoccidial, antiparasitic, immunomodulatory, anti-inflammatory, and endocrine-stimulating activities (Table 1 and Fig. 2). Plants most commonly used for phytobiotics extraction include alfalfa, bergamot, peppermint, black cumin, cayenne pepper, clove, oregano, cinnamon, and garlic (Angane et al., 2024; Ruvalcaba-Gómez et al., 2022; Sakarikou et al., 2020; Shaji et al., 2023). Natural capsaicin extracted from chili peppers has been shown to control the invasion of *Salmonella Typhimurium* into the internal organs (liver, spleen) of laying hens (Angane et al., 2024; Ruvalcaba-Gómez et al., 2022; Sakarikou et al., 2020; Shaji et al., 2023). Garlic and oregano extracts have also been clinically proven to have anti-*Salmonella* activity. Feeds containing 40 mg/mL to 80 mg/mL of garlic extract were effective in increasing the body weight of laying hens and significantly reducing mortality in groups following *Salmonella Gallinarum* infection (Angane et al., 2024; Ruvalcaba-Gómez et al., 2022; Sakarikou et al., 2020; Shaji et al., 2023). Although oregano extract shows unsatisfied efficacy in the treatment of chicken infected with *Salmonella*, adding it to the drinking water of healthy flocks can effectively prevent infections caused by *Salmonella Gallinarum* and *Salmonella Pullorum* (Angane et al., 2024; Ruvalcaba-Gómez et al., 2022; Sakarikou et al., 2020; Shaji et al., 2023).In addition to this, phytobiotics can regulate the immune system and mitigate inflammatory damage. Feed addition of a commercial phytobiotic product called Intebio can downregulate the expression of inflammatory factors IL6, IL8L2, CASP6, and IRF7 in *Salmonella Gallinarum* infected chickens, attenuating the damage caused by the inflammatory response in the early stages of the infection, and limiting the colonization of the pathogen (Angane et al., 2024; Ruvalcaba-Gómez et al., 2022; Sakarikou et al., 2020; Shaji et al., 2023). Phytobiotics, such as cinnamaldehyde and eugenol, have a similar effect, inhibiting *Salmonella* colonization in the cecum and reducing the rate of pathogen proliferation after infection (Angane et al., 2024; Ruvalcaba-Gómez et al., 2022; Sakarikou et al., 2020; Shaji et al., 2023).

### Plant essential oils

Plant essential oils belong to the generalized phytobiotics. Unlike single-component phytobiotics, essential oils are intricate blends of diverse terpenes, phenylpropenes, alcohols, acids, esters, epoxides, aldehydes, ketones, amines, and sulfides, with hydrophobic properties (Abd El-Hack et al., 2022; Adaszyńska-Skwirzyńska and Szczerbińska, 2017; Andoni et al., 2020; Angane et al., 2024; Bajpai et al., 2012; El-Saadony et al., 2022; Ruvalcaba-Gómez et al., 2022; Sakarikou et al., 2020; Shaji et al., 2023). There are more than 3000 essential oils available and most of the plant essential oils that are effective in inhibiting *Salmonella Enteritidis* belong to the family Labiatae, such as oregano leaf, white mustard, sage, and thyme. These oils mainly consist of phenols, alcohols, ketones, and aldehydes, with phenolic compounds like thymol, carvacrol, and eugenol exhibiting the highest bioactivity (Aljuwayd et al., 2023). Alcohols such as α-terpineol and nerol (Kačániová et al., 2024), ketones like carvone and camphor (Kačániová et al., 2024), and aldehydes such as nonanal, cinnamaldehyde, citral, and myrtenal also demonstrate strong antibacterial properties (Wang et al., 2023). Essential oils extracted from oregano, thyme, cinnamon, clove, mint, and camphor have shown high efficacy against *Salmonella* in clinical applications, and some of the essential oils have synergistic effects (Abd El-Hack et al., 2022; Adaszyńska-Skwirzyńska and Szczerbińska, 2017; Andoni et al., 2020; Angane et al., 2024; Bajpai et al., 2012; El-Saadony et al., 2022; Ruvalcaba-Gómez et al., 2022; Sakarikou et al., 2020; Shaji et al., 2023). These plant essential oils can alter bacterial biofilm permeability, interfere with quorum sensing mechanisms, change gut microecology (e.g., pH, redox potential), influence gene expression related to genetic adaptation (stress responses), and disrupt horizontal gene transfer and efflux pump upregulation, which induces antibiotic resistance (Abd El-Hack et al., 2022; Adaszyńska-Skwirzyńska and Szczerbińska, 2017; Andoni et al., 2020; Angane et al., 2024; Bajpai et al., 2012; El-Saadony et al., 2022; Ruvalcaba-Gómez et al., 2022; Sakarikou et al., 2020; Shaji et al., 2023).

*In vivo* and *in vitro* experiments have shown that plant essential oils, such as clove essential oil and camphor essential oil, can inhibit the rate of poultry *Salmonella* colonization in the cecum contents, assist the body's immune system in removing colonized bacteria from the cecum, and safeguard the safety of the eggs produced (Table 1 and Fig. 2). The researchers added clove or camphor essential oil at a concentration of 0.1–1.2 % to isolated chicken cecum contents containing 7.0 log10 CFU/mL of *Salmonella* and 5.0 log10 CFU/mL of *Campylobacter*, and found that most of the *Salmonella* and *Campylobacter* in the cecum contents were gradually removed. *In vivo* tests showed similar results, with feed supplementation with 0.5–1 % clove essential oil or camphor essential oil removing 50 % of *Salmonella* colonizing the cecum within two weeks (Abd El-Hack et al., 2022; Adaszyńska-Skwirzyńska and Szczerbińska, 2017; Andoni et al., 2020; Angane et al., 2024; Bajpai et al., 2012; El-Saadony et al., 2022; Ruvalcaba-Gómez et al., 2022; Sakarikou et al., 2020; Shaji et al., 2023).

The composition of essential oils depends on factors such as geographic location, soil nutrients, genetics, time of harvest, plant parts, and extraction methods, which affect the biosynthetic pathways of secondary metabolites, that correspond to different antimicrobial properties. For example, oregano leaves, belonging to the genus Origanum in the family Labiatae, a popular extracted plant in essential oils for *Salmonella* inhibition, are divided into many subtypes, such as Mexican oregano leaves (Lippia graveolens), Spanish oregano leaves (Origanumvivens), winter solstice leaves (origanum heracleoticum), sweet oregano (Origanum majorana L.) (Dantas et al., 2016). Researchers have extracted the constituents of oregano grown in different geographic locations and found that the concentration of essential oil constituents is regionally dependent, which in turn affects the antibacterial properties (Kolypetri et al., 2023; Ruan et al., 2021; Xu et al., 2022). Although plant essential oils exhibit strong antibacterial abilities, their high volatility and the thermal sensitivity, photosensitivity, and instability of many components limit their use and effectiveness (Abd El-Hack et al., 2022; Adaszyńska-Skwirzyńska and Szczerbińska, 2017; Andoni et al., 2020; Angane et al., 2024; Bajpai et al., 2012; El-Saadony et al., 2022; Ruvalcaba-Gómez et al., 2022; Sakarikou et al., 2020; Shaji et al., 2023). Together with hydrophobic properties, plant essential oils need to utilize self-emulsifying drug delivery systems to enhance bioavailability (Abd El-Hack et al., 2022; Adaszyńska-Skwirzyńska and Szczerbińska, 2017; Andoni et al., 2020; Angane et al., 2024; Bajpai et al., 2012; El-Saadony et al., 2022; Ruvalcaba-Gómez et al., 2022; Sakarikou et al., 2020; Shaji et al., 2023). Novel encapsulation techniques such as nanoparticles, microcapsules, and emulsions can increase the delivery efficiency of plant essential oils to a greater extent and improve their inhibitory effects (Abd El-Hack et al., 2022; Adaszyńska-Skwirzyńska and Szczerbińska, 2017; Andoni et al., 2020; Angane et al., 2024; Bajpai et al., 2012; El-Saadony et al., 2022; Ruvalcaba-Gómez et al., 2022; Sakarikou et al., 2020; Shaji et al., 2023). A study of nanoemulsions of Litsea cubeba essential oil suggests the possibility of applying it early on the surface of eggs to reduce horizontal transmission of *Salmonella* (Su et al., 2023). In addition, to ensure that most farms can obtain essential oils to combat *Salmonella*, the production cost of essential oils should be considered. One strategy to increase plant essential oil production and reduce costs is to mix a portion of the by-products from the essential oil extraction process. Some investigations have suggested that the byproduct of essential oil extraction, hydrosol, also exhibits certain antimicrobial properties, and it has been found that the hydrosols extracted from six different edible flowers (*Antirrhinum majus* L, *Begonia cucullata* Willd, *Calendula officinalis* L., *Dahlia hortensis* Guillaumin, *Polianthes tuberosa* L., and *Tulbaghia cominsii* Vosa) can effectively inhibit *Staphylococcus aureus* and *Salmonella enterica* (D'Amato et al., 2018; Najar et al., 2024; Tavares et al., 2022).

### Water-soluble extracts

Plant water-soluble extracts are dominated by polysaccharide complexes. Commonly used polysaccharide complexes in poultry include Atractylodes macrocephala polysaccharides, Astragali polysaccharides, Taishan horsetail pine pollen polysaccharides, alfalfa polysaccharides, and licorice polysaccharides (Zhao et al., 2023). These polysaccharide complexes consist of mannose, ribose, xylose, hexanedioic acid, glucose, fructose, rhamnose, arabinose, galacturonic acid, and glucuronic acid (Gao et al., 2020; Jiang et al., 2016; Pan et al., 2020; Zhao et al., 2023). These active ingredients can enhance the immunity and disease resistance of poultry, regulate the balance of intestinal microorganisms, and effectively alleviate various stress factors faced by poultry (Table 1 and Fig. 2). Alfalfa polysaccharides and licorice polysaccharides have demonstrated significant preventive and therapeutic efficacy against *Salmonella* in poultry. Administering alfalfa polysaccharides to *Salmonella*-infected poultry can enhance intestinal morphology and mucosal barrier function, thereby augmenting the overall resistance of the poultry. Alfalfa polysaccharides not only fostered the growth of beneficial intestinal bacteria (*Mycobacterium avium, Pasteurella, Aeromonas butyricola*, and *Puccinellidae*), but also elevated the counts of core bacteria associated with body weight (Li et al., 2021b). Simultaneously, alfalfa polysaccharides increased the expression of IgG and IgA in the serum, sIgA and sIgG in duodenal mucosa, enhanced the activity of diamine oxidase in the intestine, and upregulated the expression of tight junction proteins (claudin-1, occludin, and MUC2) in the jejunum. This acceleration facilitated the clearance of *Salmonella in vivo* (Li et al., 2021b). Analogous to alfalfa polysaccharides, licorice polysaccharides significantly reduced the counts of *E. coli* and *Salmonella* in poultry intestines, thus improving intestinal immune function and preserving a normal gut microbiota (Reda et al., 2021). Additionally, licorice polysaccharides can serve as vaccine adjuvants, notably increasing lymphocyte counts in chickens and elevating vaccine titers (Reda et al., 2021; Wu et al., 2022). In theory, other polysaccharide complexes should exhibit comparable effects against *Salmonella* infection in poultry. A study on *Salmonella typhimurium* infection in BALB/c mice revealed that *Astragalus* polysaccharides could alleviate weight loss and diarrhea induced by this pathogen (Dong et al., 2019). *Astragalus* polysaccharides notably elevated the villus height and crypt depth of the mouse jejunum, diminished inflammatory cell infiltration, and declined the expression levels of pro-inflammatory cytokines (TNF-α, IL-1β, IL-6, and IL-17) in the jejunum (Dong et al., 2019). *Astragalus* polysaccharides may exhibit similar anti-inflammatory properties in poultry infected with *Salmonella*.

### Traditional Chinese medicine compounds

Traditional Chinese medicine (TCM) compounds are essentially a combination of various natural medicines (mainly plant extracts). Unlike extracts from a single plant, TCM compounds can balance the efficacy of different components and avoid the toxic effects caused by a single medicine (Zou et al., 2024). The use of TCM compounds for poultry *Salmonella* control has been popularized in Asia (mainly in China and Korea) (Giannenas et al., 2020). Research has reported that a TCM compound called "Chuanbai likang", composed of pomegranate peel, Sanguisorba, Terminalia, Alisma, Scutellaria, honeysuckle, Phellodendron, Atractylodes, and tangerine peel, has shown significant antibacterial effects against poultry *Salmonella* (Zhang et al., 2023). The protection rate of this compound for poultry can reach 88 %∼90 % and the cure rate is 75 %∼84.4 %, which can effectively reduce the mortality rate of diseased poultry and improve the egg production rate. Similar studies proved the reliability of TCM compounds against poultry *Salmonella* (Zhang et al., 2023). A 4-flavored Chinese herbal medicine formula based on Agrimonia pilosa Ledeb, the tuber of Smilax glabra Roxb, the rhizome of Iris domestica (L.) Goldblatt and Mabb, and the root of Anemone chinensis Bunge, can significantly reduce *Salmonella Enteritidis* from poultry cecum by more than 90 % within 6h after administration (McMurray et al., 2020). Mechanistic studies have revealed that TCM compounds can significantly increase the number of B lymphocytes and enhance the body's humoral immune response to bacterial infections (Gao et al., 2022).

In addition, TCM compounds can be used in combination with probiotics or prebiotics. Research has found that herbal mixtures fermented with probiotics can effectively treat chicken white diarrhea, regulate intestinal flora, enhance immune function, and improve growth performance (Shen et al., 2024; Wang et al., 2021). However, due to the complexity of ingredient combinations and the variability in composition ratios, there is currently no precise scientific research that can clearly analyze the antibacterial mechanism of TCM compounds. Moreover, the bitter taste components in TCM compounds make them less palatable, and their direct application as feed additives may affect poultry feed intake (Song et al., 2023). Depending on the advances in metabolomics, single-cell sequencing, and neurobiology, it may be possible to clarify the antimicrobial mechanism of TCM compounds in the future, and promote their application as poultry feed additives.

## Combination of vaccines and natural medicines

### Poultry Salmonella vaccines

Although the exact role of antibodies in controlling poultry *Salmonella* infections remains unclear, clinical trials have shown that during infection, *Salmonella* attempts to suppress the number of IgG-producing plasma cells in the bone marrow by using the protein SiiE, thereby reducing serum IgG levels and escaping the humoral immune response (Jiang et al., 2023; Ruvalcaba-Gómez et al., 2022; Shaji et al., 2023). A steady increase in specific antibody IgG and IgM in the serum of poultry inoculated with SiiE-deficient strains of *Salmonella* has been reported, and the mortality rate in the takedown experiments was lower than that in the uninoculated group (Jiang et al., 2023; Ruvalcaba-Gómez et al., 2022; Shaji et al., 2023). This suggests that humoral immunity plays an important role in resistance to *Salmonella* infection. Poultry *Salmonella* vaccines commonly used in clinical practice can be divided into three categories: active attenuated vaccines, inactivated vaccines, and subunit vaccines. Due to the presence of maternally derived antibodies, *Salmonella* vaccination is usually started from 5-7 days old chicks and continued until 18-19 weeks of age (Jiang et al., 2023; Ruvalcaba-Gómez et al., 2022; Shaji et al., 2023). Commonly used active attenuated vaccines are *Salmonella Gallinarum* 9R strain (Nobilis® SG 9R) and Salenvac® (Merck, Rahway, NJ, USA) (Jiang et al., 2023; Ruvalcaba-Gómez et al., 2022; Shaji et al., 2023). These two vaccines can provide effective immunoprotection for chicks, but there is a possibility of virulence resuscitation, which has the risk of further increasing the environmental contamination of pathogenic bacteria and jeopardizing the health of the population. Inactivated vaccines, on the other hand, are usually considered safe, but they often fail to elicit adequate protection against the disease. Subunit vaccines, which are now the hotspot of clinical research and development, consist of proteins, polysaccharides, or peptides with antigenic properties on the surface of *Salmonella* organisms, such as SseB, FliC, OmpD, OmpC, and PagN subunit vaccines (Jiang et al., 2023; Ruvalcaba-Gómez et al., 2022; Shaji et al., 2023). Subunit vaccines are effective in eliciting the immune response of the body and, unlike active attenuated or inactivated vaccines that contain intact pathogens, they do not carry the risk of causing disease and have a higher safety profile for clinical application.

### Enhance the protection of vaccines with natural medicines

Vaccines are an important means of preventing *Salmonella* infections. However, they are not 100 % effective in controlling *Salmonella* colonization in the chicken intestine (Groves et al., 2021). The composition of beneficial gut microbiota affects the immune response after vaccination. Clinical practice has demonstrated that feeding natural medicines (primarily probiotics, prebiotics, and organic acids) to poultry before or after vaccination can enhance the protection of the vaccine (Bajagai et al., 2016; El-Shall et al., 2019; Groves et al., 2021; Prado-Rebolledo et al., 2017; Praharaj et al., 2015; Redweik et al., 2020b). The immune control of *Salmonella* infection in the chicken gastrointestinal tract mainly relies on cellular and humoral mediated immunity. Before sexual maturity, the chicken's cellular immune organs are functionally active and intact. During this stage, vaccines, which are thought to provoke only a humoral mediated immune response, can work synergistically with the cellular immune system to prevent *Salmonella* infection or eliminate pathogenic microorganisms in the gastrointestinal tract within several weeks after infection (Bailey et al., 2007; Groves et al., 2021; Johnston et al., 2012; Wigley et al., 2005). However, after sexual maturity, thymic involution weakens the cellular mediated immune response. Vaccines alone cannot prevent the re-colonization of *Salmonella*, leading to infections in laying flocks and egg contamination (Bailey et al., 2007; Groves et al., 2021; Johnston et al., 2012; Wigley et al., 2005). Supplementing organic acids (e.g., propionic acid, butyric acid, and formic acid) or probiotic combinations can modulate the gut microbiota, creating an environment unfavorable for *Salmonella* growth (Bajagai et al., 2016; El-Shall et al., 2019; Groves et al., 2021; Prado-Rebolledo et al., 2017; Praharaj et al., 2015; Redweik et al., 2020b). This helps maintain effective protection against pathogens during sexual maturity, a period when the cellular immune system is suppressed and degenerates. In addition, the chicken cecum is a major site of neurochemical metabolism. Gut microbiota can influence brain function by regulating tryptophan metabolism and serotonin synthesis (Redweik et al., 2020a). Some gut microbes can synthesize and metabolize neurotransmitters like γ-aminobutyric acid (GABA), catecholamines, acetylcholine, and others, thereby affecting neural function (Redweik et al., 2020a). Feeding probiotics, prebiotics, or other natural compounds can improve the gut microbiota and promote the synthesis and secretion of excitatory neurotransmitters in the gut (Redweik et al., 2020a). These neurochemical compounds can help alleviate adverse neurological reactions post-vaccination, such as stress and reduced feed intake. Recent studies have demonstrated that the combination of probiotics with live attenuated vaccines can enhance growth performance and reduce mortality, thus limiting the colonization of *Salmonella* in chicken (Bajagai et al., 2016; El-Shall et al., 2019; Groves et al., 2021; Prado-Rebolledo et al., 2017; Praharaj et al., 2015; Redweik et al., 2020b).

## Delivery systems of natural medicines

### In ovo injection technology

Although natural medicines have the potential to replace antibiotics for the prevention and treatment of *Salmonella* infection, their slow onset of action makes it difficult for poultry, especially newborn chicks, to establish a complete immune barrier during the critical window period. To address this shortcoming, clinical practices have proposed the use of *in ovo* injection techniques to enhance the immunity of newborn chicks (Givisiez et al., 2020). Based on the injection site, *in ovo* injection techniques can be categorized into three types: yolk sac injection, allantoic injection, and amniotic cavity injection. Amniotic and allantoic injections are often used for pre-hatch vaccination against *Salmonella* (Table 1 and Fig. 2) (Givisiez et al., 2020). It has been shown that injecting a cytosine-phosphate-guanine oligodeoxynucleotide (CpG-ODN) vaccine into the allantoic cavity on the 18th day of incubation can reduce the colonization of highly pathogenic and antibiotic-resistant *Salmonella Heidelberg* (SH) strains in the cecal contents during the first week of post-infection, thereby improving the intestinal health condition of chicks (Alves et al., 2024). Natural drugs are more suitable for yolk sac injections due to the larger injectable dose and the presence of fat-soluble components (Peebles, 2018). Moreover, the yolk sac is larger in volume, with a lower density of blood vessels, making it easier to operate and safer than amniotic cavity injection (Peebles, 2018). In the late stages of incubation, chicken embryos primarily absorb nutrients from the yolk sac, which also facilitates the rapid absorption of natural medicines. Clinical studies have demonstrated that *in ovo* injection of natural medicines (such as probiotics, synbiotics, postbiotics, plant antibiotics, and water-soluble polysaccharides) helps to against *Salmonella* infections of newborn chicks (Cox and Dalloul, 2015; De Oliveira et al., 2014; El-Kholy et al., 2021; Li et al., 2021a; Madej et al., 2015; Oladokun et al., 2023; Ruvalcaba-Gómez et al., 2022). Although the mechanisms of this strategy are not fully understood, it is clear that *in ovo* injection of natural medicines helps improve the composition and structure of the embryonic gut microbiota, directly or indirectly controlling the colonization of *Salmonella* in the intestine, while inducing the innate immune system to clear pathogens (De Oliveira et al., 2014). Combined injection of nutrients, hormones, vaccines, and immune stimulants can provide better control of *Salmonella* in newborn chicks by improving nutrient absorption, rapid development of jejunal villi, stimulation of the immune system, increased expression of enzymes and transit proteins, and enhancement of resistance to pathogens as well as promotion of early development of the digestive tract and muscular tissues (Peebles, 2018).

### Nanoparticle-based delivery systems

Natural medicines, especially plant essential oils, require specific delivery systems to improve their bioavailability. The main reasons for this are: 1) chemical instability of plant essential oils. The active components in essential oils, such as terpenes, phenylpropanoids, esters, epoxides, and aldehydes, are prone to oxidation or decomposition when exposed to factors like heat, humidity, oxygen, or light (da Silva Gündel et al., 2020; Dupuis et al., 2022). 2)rapid metabolism of plant essential oils. Pharmacokinetic analyses show that plant essential oils are absorbed quickly after oral, pulmonary, or dermal administration, reaching peak levels within 2 h of administration. However, the chemical components of plant essential oils are no longer detectable in the blood within 5 h (Dupuis et al., 2022; Horky et al., 2019). Nanoparticles containing plant essential oils (EOs NPs) are currently one of the effective solutions to improve their delivery, release, and bioavailability in tissues and cells (Shin et al., 2019). The main types of plant essential oil nanoparticles used in clinical applications are chitosan, cellulose, zeinolysin, sodium alginate, PLGA, and lipid-based systems (Table 2 and Fig. 2). Based on their chemical composition, these nanoparticles can be classified into two major categories: polymeric and lipid-based nanoparticles. Both of them are biodegradable and environmentally friendly. Among them, chitosan nanoparticles and their derivatives have been most widely used in the past decade for the drug delivery of plant essential oils (Dupuis et al., 2022). Chitosan nanoparticles containing plant essential oils can open tight junctions in intestinal cells to allow for better essential oil absorption and transport essential oils to the surface of bacterial cell membranes to inhibit their proliferation (Nouri, 2019). In contrast, pure essential oils are unable to effectively penetrate the cellular layer of the intestinal wall and make contact with bacterial cell membranes due to their low water solubility (Mohammadi et al., 2020). In addition, chitosan itself has an antibacterial effect. The interaction between the positively charged amino group of chitosan and the negative charge on the surface of microbial cell membranes can reduce the permeability of bacterial cell membranes, change the electron transport chain of bacterial membranes, and inhibit bacterial colonization (Hosseini and Meimandipour, 2018; Nouri, 2019; Rozman et al., 2020). Numerous clinical practices have demonstrated the effectiveness of chitosan in delivering plant essential oils. It can be used as a feed additive in poultry production, adhering to gastrointestinal mucosa for drug delivery and providing broad-spectrum antibacterial activity (Amiri et al., 2021; Hosseini and Meimandipour, 2018; Jamil et al., 2016; Mohammadi et al., 2020; Nouri, 2019; Rozman et al., 2020). This helps increase poultry feed intake and conversion rates, while enhancing the immune system's ability to resist pathogenic infections (Amiri et al., 2021; Hosseini and Meimandipour, 2018; Jamil et al., 2016; Mohammadi et al., 2020; Nouri, 2019; Rozman et al., 2020).

**Table 2:** Characteristics of nanomaterials for delivery of essential oils.

**Types**	**Composition**	**NPs size (nm)**	**Reported EOs-loaded**	**Advantages**	**Drawbacks**	**References**
Chitosan	Medium molecular weight chitosan	181-276	Mint, thyme, cinnamon, cardamom, garlic, anise, oregano, pennyroyal, green tea, etc.	Non-toxic, renewable, mucoadhesive, good permeability, innate antimicrobial potential, low cost, biodegradable, high tensile strength, hemostatic.	No major drawbacks	(Bagheri et al., 2021; Dupuis et al., 2022; Nouri, 2019; Rozman et al., 2020)
Zein	Zein from maize	139-145	Thyme, clove, garlic, oregano, carvacrol, etc.	Biocompatible, high encapsulation efficiency, low cost, ability to form a film.	Need toxicological studies to prove its safety.	(de Freitas Proença et al., 2024; Dupuis et al., 2022; Gonçalves da Rosa, 2020)
Cellulose	Cellulose nanofibers	210-338	Thyme, santolina, pepper tree, eucalyptus, etc.	Low cost, biodegradable, non-toxic, mucoadhesive, hemostatic, high water absorption, high retention capacity.	No antimicrobial properties on its own, critical debate about its toxicology assessment.	(Dupuis et al., 2022; Simsek et al., 2020)
NaAIg	Alginic acid sodium	-	Chamomile, cinnamon, lavender, tea tree, peppermint, eucalyptus, lemongrass, allspice, black pepper, etc.	Biocompatible, high water absorption, non-toxic, mucoadhesive, hemostatic.	No antimicrobial properties on its own.	(Dupuis et al., 2022; Wang et al., 2023)
PLGA	PLGA with a copolymer of DL-lactide and glycolide	179-192	Black caraway, clove, cinnamon, anise, lemongrass, fennel, pennyroyal, thyme, oregano, etc.	Biodegradable, biocompatible, low toxicity.	High cost, difficulty to scale-up, low entrapment efficiency.	(Dupuis et al., 2022; Nguyen et al., 2022)
Solid lipid	Fatty acids or mono-, di-, or triglycerides	800-828	Eucalyptus, rosemary, clove, etc.	Low toxicity, can be produced on a large scale, biodegradable, able to transport hydrophobic substances.	Limited solubility in water, oxidation of lipids, poor encapsulation of EOs, release of EOs during storage.	(Dupuis et al., 2022; Fazly Bazzaz et al., 2018)
Nanoemulsion	Transparent and translucent oil in water emulsions	20-200	Lemongrass, eucalyptus, winter savory, thyme, oregano, mint, cinnamon, carvacrol, eugenol, etc.	Low cost, ease of preparation, physical and thermodynamic stability, non-toxic.	Limited solubility in water, oxidation of lipids, poor encapsulation of EOs, release of EOs during storage.	(Dupuis et al., 2022; Ibrahim et al., 2021)
Liposome	Enclosed spherical vesicles with one or several concentric phospholipidic bilayers and an internal aqueous phase	204-380	Clove, bunge pricklyash pericarp, laurel, etc.	Non-immunogenic, biocompatible, interesting approach to incorporate EOs and to improve their solubility.	Rapid release of the entrapped drug, high cost, poor loading efficacy, instability.	(Cui et al., 2017; Dupuis et al., 2022; Eldiasty et al., 2024; Meligy et al., 2023)
Nanostructured lipid carriers	Similar with solid lipid, emergent generations of lipidic nanoparticles	107-238	Tea tree, peppermint, pennyroyal, etc.	Biocompatibility, efficacy, safety, able to transport lipophilic and hydrophilic substances, increases stability and bioavailability of the substance, good bio-adhesion and penetration through the lipid bilayer, synergistic action when combined with EOs for antimicrobial properties.	No major drawbacks	(Dupuis et al., 2022; Khezri et al., 2020)
Niosome	Nontoxic self-assembly vesicles with a single or multiple layered structure	-	Rosemary, thyme, savory, oregano, etc.	Easy to store and handle, low toxicity, able to transport hydrophobic and hydrophilic substances, biodegradable.	No major drawbacks	(Abonashey et al., 2024; Dupuis et al., 2022)

NPs: nanoparticles; EOs: essential oils.

## Conclusions

Although conventional antibiotics can quickly and effectively treat *Salmonella* infections in poultry, the induced resistant pathogens and issues related to environmental residues pose potential safety risks. To address these shortcomings, clinical practices have gradually shifted towards developing and promoting new natural medicines for preventing and treating *Salmonella* infections in poultry. Probiotics, prebiotics, essential oils, phytobiotics, and herb medicine compounds have shown promise in combating *Salmonella* infections in poultry. These natural medicines can effectively enhance the intestinal microenvironment, increase the feed intake of infected chickens, promote weight gain, and improve immunity and pathogen clearance rates. Through advanced drug purification technology, nanoparticle delivery systems, and *in ovo* injection technology, the onset time, pharmacokinetic properties, and *in vivo* metabolic efficiency of natural medicines have been significantly improved. It is expected that in the near future, more high-quality and effective natural medicine products against poultry *Salmonella* infections will be available for clinical use.

## Author contributions

YZ: Conceptualization, Investigation, Visualization, Writing–original draft, Writing–review and editing. JL: Visualization, Writing–original draft. YP: Visualization, Investigation. KS: Investigation, Writing–review and editing. PM: Investigation. XL: Funding, Investigation. SS: Conceptualization, Supervision, Funding, Writing–review and editing.

## Ethics statement

This research did not involve live animal experiments.

## Declaration of competing interest

The authors declare that they do not have any competing interests. There were no financial and personal relationships with other people or organizations that could have inappropriately influenced the study. There were no professional or other personal interests of any nature or kind in any product, service and/or company that could be construed as influencing the position presented in, or the review of, the manuscript entitled.
